# Effects of hormone-primed oviduct epithelial cell co-culture system on swine SCNT embryo development

**DOI:** 10.3389/fcell.2025.1692877

**Published:** 2025-10-09

**Authors:** Zhong-Ping Chen, Jian Wang, Chang-Di Bian, Dong-Yue Wang, De-Hui Feng, Ming-Yi Wei, Da-Wei Yu, Wei-Jun Sun, Lin-Lin Zhang

**Affiliations:** ^1^ Tianjin Key Laboratory of Agricultural Animal Breeding and Healthy Husbandry, College of Animal Science and Veterinary Medicine, Tianjin Agricultural University, Tianjin, China; ^2^ Tianjin Keylink Biotechnology Co., Ltd, Tianjin, China; ^3^ Institute of Animal Science, Chinese Academy of Agricultural Sciences, Beijing, China

**Keywords:** swine embryo development, co-culture, oviduct epithelial cells, PI3K, SMART-seq2

## Abstract

The developmental efficiency of swine somatic cell nuclear transfer (SCNT) embryos remains limited, primarily due to the lack of physiologically relevant *in vitro* culture conditions that can fully support reprogramming and early embryogenesis. In this study, we established a co-culture system using swine oviduct epithelial cells (OECs), including untreated and hormone-pretreated OECs with estradiol and progesterone (EP-OECs), to better mimic the oviductal environment. Compared with the control group, EP-OECs exhibited elevated expression of the oviduct-specific marker OVGP1. Moreover, SCNT embryos co-cultured with EP-OECs displayed a significantly higher blastocyst formation rate (control: 18.6% ± 0.01; OEC: 24.5% ± 0.01; EP-OEC: 30.5% ± 0.03). Although the total blastocyst cell number did not increase, co-culture significantly elevated intracellular glutathione (GSH) levels and reduced oxidative stress at key developmental stages. Single-cell transcriptomics (SMART-seq2) analysis revealed that the co-culture activated multiple metabolic pathways, including the pentose phosphate pathway and lipid metabolism, thereby improving redox regulation and energy utilization. Moreover, embryonic development was enhanced through the modulation pluripotency-associated factors, including *SOX2*, and activation of the PI3K–AKT signaling cascade. Notably, OEC co-culture induced *PI3K* upregulation at the 8-cell stage and further affected *PDK* expression. OEC/EP-OEC co-culture treatment suggests PI3K-AKT signaling pathway activation in embryos, which may be a key mechanism promoting embryonic development. These findings indicate that hormone-primed OEC co-culture provides a microenvironment that closely resembles *in vivo* conditions, offering an effective strategy for improving the efficiency of swine cloning and a new insight into the molecular mechanisms underlying embryonic development *in vitro*.

## Introduction

Somatic cell nuclear transfer (SCNT) is a cloning strategy in which the nucleus of a differentiated somatic cell is introduced into an enucleated oocyte, allowing the donor genome to be reprogrammed and support full embryonic development. This technique has wide-ranging applications, including the generation of genetically modified animals, preservation of endangered species, and investigation of developmental and epigenetic mechanisms. Among livestock species, swine have recently gained particular prominence as an experimental model owing to their close anatomical and physiological resemblance to humans, which enhances their value in both agricultural and biomedical research. Nevertheless, the efficiency of swine SCNT remains lower than that achieved in mice and falls short of the levels observed in cattle and sheep, primarily due to incomplete nuclear reprogramming and suboptimal *in vitro* culture conditions (Inoue, 2023; Whitworth and Prather, 2010; Vazquez-Avendaño et al., 2022; Akagi et al., 2014). Despite these constraints, advances in swine SCNT have facilitated the generation of transgenic swine, underscoring their enormous potential in agricultural breeding, xenotransplantation, and disease modeling (Tanihara et al., 2021).

Compared with *in vivo*-derived embryos, SCNT embryos are more fragile and exhibit reduced survival capacity after implantation (Lin et al., 2015). The low efficiency of *in vitro* embryo development is largely because current handling and culture systems fail to adequately mimic the *in vivo* environment (Gualtieri et al., 2024). This difference in culture conditions may lead to inaccurate epigenetic reprogramming of somatic cells in cloned embryos (Song et al., 2014). To better mimic the *in vivo* environment, researchers have used co-culture to simulate the *in vivo* environment during *in vitro* embryo culture (Schoen and Chen, 2018). Previous studies have demonstrated that co-culture with oviduct epithelial cells (OECs) can enhance embryonic development in canine, human, and sheep species. The infundibulum and the ampulla oviduct epithelial cells positively influence the meiotic resumption and progression of canine oocytes (Bogliolo et al., 2002). Co-culture of human embryos with oviductal cells may improve embryonic development *in vitro*; the percentage of hatching blastocysts was significantly higher for embryos co-cultured from day 1 post-insemination (38%) than for embryos that had not been co-cultured (7%) (Yeung et al., 1992). In the *in vitro* embryo culture of sheep, the highest percentage (72.45%) of the cleavage occurred in zygotes co-cultured with oviduct epithelial cells (Ondho et al., 2020). Several secretory epithelial cells within the oviduct provide the necessary environmental and nutritional support for early embryonic development (Sidrat et al., 2020). OECs secrete a complex mixture of proteins, amino acids, carbohydrates, lipids, and extracellular vesicles on the mucosal surface. This secretion influences key processes, including sperm capacitation, zona pellucida remodeling, and embryonic genome activation (Panyarachun et al., 2021). Moreover, the oviductal fluid facilitates early dialog between the embryo and mother through intercellular contact, secreted molecules, and extracellular vesicles (Fang et al., 2022).

Hormonal pretreatment of OECs with estrogen and progesterone can induce morphological and secretory changes similar to those that occur in the endocrine environment *in vivo* (Du et al., 2023). Furthermore, comparing estradiol (E2)- and progesterone (P4)-treated OECs (EP-OECs) with untreated OECs provides a valuable framework for investigating the role of hormonally regulated anatomical changes in somatic cell–embryo communication. The proposed mechanisms underlying this enhancement include (1) the reduction of oxidative stress through antioxidant and reactive oxygen species (ROS) scavenging enzyme secretion, (2) provision of optimal metabolic substrates, (3) molecular chaperone release that mitigates environmental stress, and (4) extracellular vesicle delivery containing regulatory RNA and proteins (Wu et al., 2024; Meixiang et al., 2023; Dissanayake et al., 2021). However, the precise mechanisms involved in pig development, particularly regarding SCNT-derived embryos under hormone-regulated co-culture conditions, remain largely unresolved.

In this study, we investigated the effects of co-culturing EP-OECs with OECs on the early embryonic development of SCNT embryos. The cleavage rate, blastocyst formation rate, and intracellular ROS and GSH levels were quantified. Single-cell transcriptomics has significantly advanced the study of developmental regulation in oocytes and early embryos (Yan et al., 2013; Asami et al., 2022; Santana et al., 2019). Using this approach, SMART-seq2 technology was used to enable transcriptomic profiling at a single-embryo resolution, examining the impact of co-culture on embryonic transcriptional dynamics and gene expression patterns to identify key regulatory pathways and candidate genes. The novelty of this study lies in the integrated application of hormonally pretreated OEC co-culture and single-embryo transcriptomic profiling, which together offer mechanistic insights into how endocrine-modulated oviductal microenvironments affect the developmental competence of porcine SCNT embryos.

## Materials and methods

### Ethical approval

Ethical approval was granted by the Experimental Animal Ethics Committee of Tianjin Agricultural University (No. 2025LLSC53).

### Experimental design

Three experiments were performed to investigate the effects of OECs on the preimplantation development of SCNT embryos. First, primary OEC cell lines were established from the ampulla to the isthmus of the fallopian tube. Two types of OECs were cultured: E2- and P4-treated OECs (EP-OECs) and hormone-free OECs. EP-OECs and OECs were characterized by immunofluorescence staining, Western blotting, and quantitative real-time polymerase chain reaction (RT-qPCR). Second, the co-culture of EP-OECs and OECs with SCNT embryos was examined to assess their impact on embryonic developmental potential. Embryo developmental potential and quality were evaluated based on the cleavage rate, blastocyst formation rate, hatching rate, total cell number, and cellular ROS and GSH levels. Finally, single-cell smart-seq sequencing was performed on embryos during co-culture to elucidate the underlying mechanisms of the co-culture system in embryo development.

### Swine OEC isolation and culture

Eight clinically healthy, large white sows from the Keylink Co., Ltd. (Tianjin, China) pig farm were selected for estrus synchronization treatment, six of which were used for salpingectomy (3–5 days post-ovulation), and two of which were used for artificial insemination and *in vivo* blastocyst retrieval. The samples were maintained at 36 °C in saline containing 3% penicillin–streptomycin and were transported to the laboratory within 2 h of collection. After rinsing, the tissues were sliced into 1 mm^3^–3 mm^3^ blocks and evenly inoculated into culture bottles measuring 25 cm^2^. The blocks were added to 0.5 mL Dulbecco’s modified Eagle medium containing 1% penicillin–streptomycin solution, 5 ng/mL EGF, and 15% fetal bovine serum (FBS) with immersion blocks used to prevent the tissue mass from floating. Next, the blocks were cultured in an incubator, and the culture medium was replaced daily. After adhering to the wall for 60 h, the tissue blocks were carefully removed, and the culture medium was changed for subsequent incubation. The medium was changed once every 3 days until the monolayer cells covered approximately 90% of the area. The cells were treated, digested with 0.25% trypsin, and passaged. Following the reports of Du et al. (2023), OECs were treated with 50 pg/mL E2 and 0.5 ng/mL P4 for 12 h, and the cells were detached via trypsin treatment and collected for subsequent experiments.

### Immunofluorescence staining

OECs, EP-OECs, and swine fetal fibroblasts (PFFs, negative control) were characterized by immunocytochemistry for OVGP1 protein expression. In brief, cells were warmed and seeded on 14-mm coverslips in 24-well culture plates (at a density of 3 × 10^5^ cells/cover) and cultured as previously described (Zhao et al., 2022). The culture medium was renewed every 48 h until the cells were semi-confluent. Once a semi-confluent monolayer was obtained, the cells were washed twice with phosphate-buffered saline (PBS) and fixed with 4% paraformaldehyde for 15 min. Following fixation, cells were permeabilized with 0.5% Triton-X 100 in PBS. Cells were blocked with 5% goat serum for 30 min at room temperature. After blocking, cells were incubated with anti-OVGP1 (Proteintech, 22324-1-AP) for 1 h at room temperature. After washing with PBS, the sections were incubated with CoraLite488-conjugated Goat Anti-Rabbit IgG (H + L) (Proteintech, SA00013-2) for 1 h at room temperature. For Hochest33342 staining, cells were incubated with Hochest33342 at 37 °C for 10 min and then observed and photographed using a Leica inverted fluorescence microscope (Leica DMI6000 CS, Germany).

### Western blotting

Cells were collected in RIPA buffer supplemented with a protease inhibitor. The loading buffer was added, and the samples were heated in boiling water for 10 min. Equal amounts of protein (80 µg) were fractionated by SDS-PAGE and transferred to a polyvinylidene fluoride (PVDF) membrane (ISEQ00010, Millipore). Diluted samples were loaded onto a 15% polyacrylamide gel, and electrophoresis was performed for 3 h at a constant voltage (140 V) at room temperature. The membranes were then transferred onto Merck Millipore PVDF membranes. The PVDF membrane was blocked with skimmed milk for 1 h and incubated overnight with OVGP1 polyclonal antibody (Proteintech, 22324-1-AP) and β-actin mouse monoclonal antibody (Utibody, um4001) at 4 °C in a two-dimensional shaker. After washing thrice with TBS-T solution, membranes were exposed to goat anti-rabbit IgG H&L (Proteintech, RGAR001) and goat anti-mouse IgG H&L (Proteintech, RGAM001) for 1 h at room temperature. After washing thrice with 1x TBST, the immunoblots were visualized using ECL solution (Biosharp, BL520B) on a Tanon 5200 Image Analyzer (Tanon, Shanghai, China) and analyzed using NIH ImageJ software.

### RT-qPCR

Total RNA was extracted by nano-magnetic beads using a MagBeads Total RNA Extraction Kit (DP761, TIANGEN, China). A FastKing One Step RT-qPCR Kit was used to perform RT-qPCR (FP313, TIANGEN, China). Glyceraldehyde-3-phosphate dehydrogenase was used as an internal reference to evaluate *OVGP1*, estrogen receptor 1 (*ESR1*), and progesterone receptor (*PGR*). All primer sequences used in this experiment are listed in Table 1. The RT-qPCR analysis was performed using a CFX96 Touch Real-Time PCR Detection System (Bio-Rad, Hercules, CA, United States). RT-qPCR results were analyzed using the 2^−ΔΔCT^ method.

**TABLE 1:** Information on the primer sequences.

Gene	Primer Sequence (5'->3′)		Anneal temperature (°C)
	Forward	Reverse	
*GAPDH*	TCGGAGTGAACGGATTTGGC	TGACAAGCTTCCCGTTCTCC	60
*OVGP1*	GAAGGAGTGGGTCGGCTATG	GCTAACCTCAGCCTTGAGCA	60
*ESR1*	AGGGAAGCTCCTGTTTGCTC	CCAGAGACTTCAGGGTGCTG	60
*PGR*	GTGTCCTTACCTGTGGGAGC	TTCTAAGGCGACAAGCTGGG	60

### SCNT embryo preparation

Swine ovaries were obtained from pre-pubertal gilts at a local slaughterhouse, placed in 0.9% saline with 2% penicillin–streptomycin at 37 °C, and transported to the laboratory within 4 h. Cumulus–oocyte complexes with multiple layers of cumulus cells were selected from anterior follicles (3 mm–6 mm) and cultured in the maturation medium at 38.5 °C and 5% CO_2_ in air during the initial phase (0 h–22 h). The medium used for *in vitro* maturation was modified tissue culture medium 199 (TCM-199, Gibco, Thermo Fisher Scientific, Waltham, MA, United States) supplemented with 10% swine follicle fluid, 0.57 mM L-cysteine, 10 ng/mL EGF, 0.91 mM sodium pyruvate, 10 IU/mL pregnant mare serum gonadotropin (PMSG), 10 IU/mL human chorionic gonadotropin (HCG), and 75 μg/mL kanamycin. After the second phase (22 h–44 h, PMSG- and HCG-free), cumulus cells were removed using 0.1% hyaluronidase, and oocytes with the first polar body extrusion were regarded as metaphase II (MII) oocytes. MII oocytes in the manipulation medium (M199 with 2% FBS) were enucleated by removing the first polar body and a small amount of the surrounding cytoplasm, followed by a single donor cell injection into the perivitelline space. Reconstructed embryos were activated in the activation medium (280 mM mannitol, 0.01 mM CaCl_2_, 0.01 mM MgCl_2_, 0.05 mM HEPES, and 1% BSA) at 130 kV/cm, 100 µs twice. After activation, successful membrane fusion was confirmed by observing the fused oocytes under a stereomicroscope, and embryos were washed and transferred into swine zygote medium-3 (PZM-3) medium at 38.5 °C in a 5% CO_2_ incubator (Zhang et al., 2022). The cleavage and blastocyst rates were determined at 48 and 168 h, respectively. The total blastocyst cell number was counted using h33342 staining. Blastocysts were stained for 5 min in PBS containing 10 μg/mL Hoechst 33342 and 0.1% BSA, mounted on glass slides in 100% glycerol droplets, and gently covered with a coverslip. Stained blastocysts were examined under a fluorescence microscope (Leica DMI6000 CS, Germany).

### Determination of intracellular ROS and GSH levels

The endogenous ROS and GSH levels in 2–4 cells, 8 cells, and blastocysts were assessed using 2′,7′-dichlorofluorescein (H2DCFDA, HY-W040143, MCE) and 4-chloromethyl-6,8-difluoro-7-hydroxycoumarin (CMF2HC, HY-D1571, MCE), respectively. Washed embryos were incubated with H2DCFDA and CMF2HC for 30 min in the dark. Images were taken using a Leica DMI6000 fluorescence microscope with filters that allowed the detection of ROS and GSH measurements at 496 and 405 nm, respectively. The fluorescence intensity was analyzed using ImageJ software.

### Smart-seq sequencing

Typically, 2–4 cell, 8 cell, and blastocyst embryo samples were collected from the control, EP-OEC, and OEC groups. Each group of experiments was repeated three times. Each time, three embryos were used, which best represented the average level of the group. A total of 27 embryos were used in the 2–4 cell, 8-cell, and blastocyst stages. Before sample collection, the embryos were exposed to a 0.5% pronase solution for 30 s to remove the zona pellucida. Subsequently, the embryo samples were washed thrice with calcium- and magnesium-free DPBS and transferred into a microcentrifuge tube containing lysis buffer before being stored at −80 °C for subsequent analysis. RNA-Seq was performed using the SMART-Seq II. In brief, total RNA samples were mixed with oligo-dT and deoxyribose nucleoside triphosphates. Reverse transcription with oligo-dT primers specifically annealed to the polyadenylated (poly-A) tails of mRNA molecules, ensuring selective capture of mRNA during complementary DNA (cDNA) synthesis. The template was changed at the 5′ end of the RNA, and the full-length cDNA was amplified via PCR, after which the PCR products were purified and selected using the Agencourt AMPure XP-medium kit. After purification, amplified cDNA was used for library construction. The PE150 double-end sequencing program was run on the NovaSeq sequencing platform. Raw sequencing data were processed as previously described (Shalek et al., 2014). Short sequencing reads were aligned to the Sscrofa11.1 genome using RSEM (version 1.2.1) to quantify gene expression levels (transcripts per million, TPM) for all Sscrofa11.1 genes in all samples. Cells with fewer than <1,000 detected unique genes were filtered out from the dataset. The data were log-transformed (log TPM +1) for all downstream analyses. Kyoto Encyclopedia of Genes and Genomes (KEGG) enrichment analysis was performed using TBtools (Zhang et al., 2023).

### Statistical analyses

Each experiment was performed at least three times, and the results are presented as the mean ± standard error of the mean. Statistical analyses were conducted using GraphPad Prism software (version 9.0). Data (excluding sequencing data) were analyzed using repeated measures analysis of variance (ANOVA) (RM ANOVA) or one-way ANOVA when appropriate. Statistical significance was set at *p* < 0.05, unless otherwise stated.

## Results

### Characterization of OECs

**FIGURE 1:**
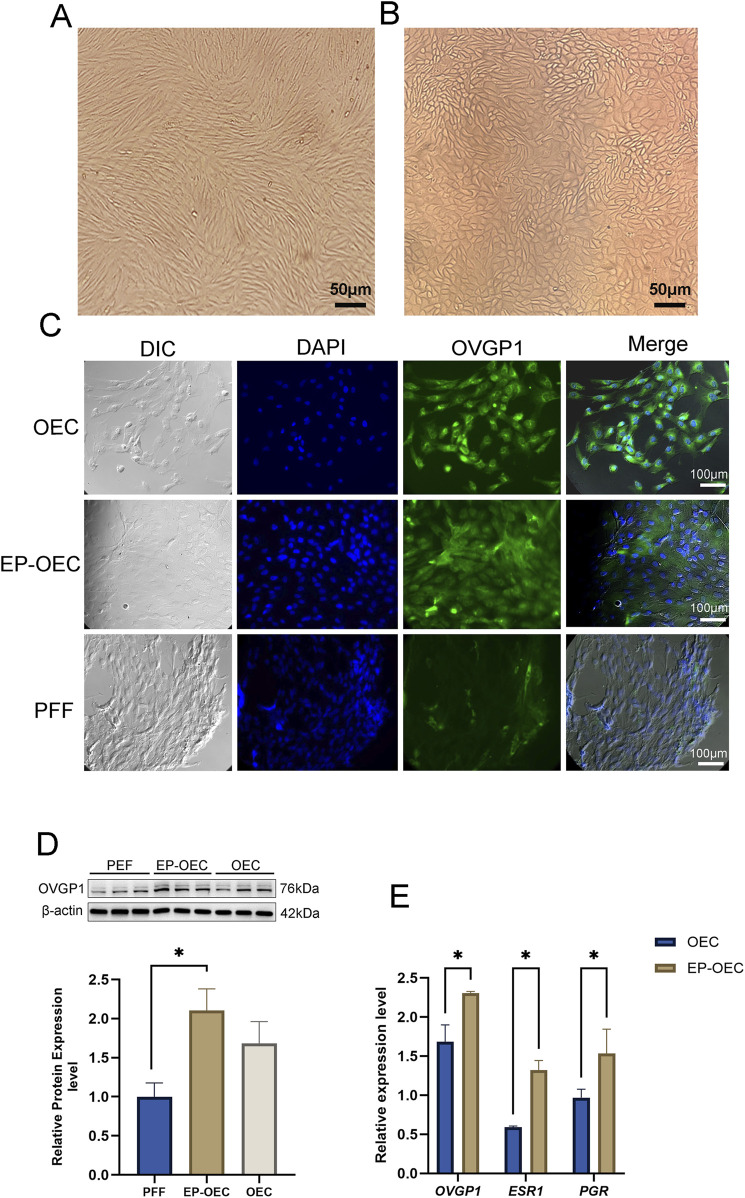
Characterization of EP-OECs and OECs. **(A)** Morphology in swine oviduct epithelial cells; scale bar, 50 μm. **(B)** Morphology in swine fetal fibroblasts; scale bar, 50 μm. **(C)** Immunofluorescence staining of OVGP1 (green) in EP-OECs and OECs, with nuclei counterstained with Hoechst 33,342 (blue). PFFs served as the negative control. Scale bar, 100 μm. **(D)** Western blot analysis of OVGP1 expression in EP-OECs and OECs, with β-actin as the loading control. **(E)** RT-qPCR analysis of *OVGP1*, *ESR1*, and *PGR* mRNA levels in EP-OECs and OECs. GAPDH served as the reference gene. The data are indicated as the mean ± SEM. *p < 0.05, **p < 0.01, and ***p < 0.001. EP-OECs, estradiol and progesterone-treated oviduct epithelial cells; OECs, oviduct epithelial cells. PFFs, swine fetal fibroblasts.

To characterize OECs based on cell morphology, we compared the morphologies of PFFs and OECs. Unlike fusiform PFF, OEC morphology is polygonal with a highly granular cytoplasm, which is the unique morphology of epithelial cells (Figures 1A, B). Additionally, immunofluorescence exhibited positive labeling for OVGP1, indicating the correctness of the origin of the fallopian tube (Figure 1C). The PFF group, used as a negative control, was OVGP1-negative. Moreover, we observed that the immunofluorescence intensity of OVGP1 in EP-OECs was significantly higher than that in OECs, which was confirmed by Western blotting (Figure 1D). Additionally, the expression levels of the OEC-related genes *OVGP1*, *ESR1*, and *PGR* were analyzed by RT-qPCR. The results revealed that the expression levels of these genes in EP-OECs were significantly higher than those in OECs (Figure 1E), indicating that hormone treatment promoted *OVGP1*, *ESR1*, and *PGR* expression.

### Effect of OEC co-culture on SCNT embryo development

**FIGURE 2:**
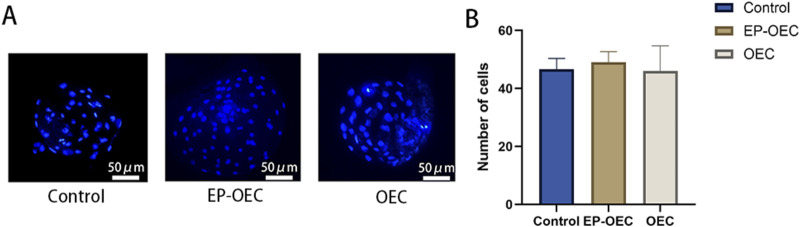
Blastocyst cell number analysis. **(A)** Representative images of DAPI-stained nuclei in blastocysts from control, EP-OEC, and OEC groups. Scale bar, 50 μm. **(B)** Quantification of the total cell numbers per blastocyst. Quantified data show the means of standard deviations. Control, SNCT embryos without co-culture treatment. EP-OECs, SCNT embryos co-cultured with oviduct epithelial cells treated with estradiol (E2) and progesterone (P4). OECs, SCNT embryos co-cultured with oviduct epithelial cells without hormone treatment.

To assess the effect of OEC co-culture on *in vitro* embryo development, we compared the cleavage and blastocyst formation rates among the control, EP-OEC, and OEC groups (Table 2). The cleavage rate in the EP-OEC group was significantly higher than that in the control group. Simultaneously, the OEC group exhibited an increase compared with the control group, although the difference was non-significant. The blastocyst formation rate was significantly improved by co-culture, with the highest increase observed in the EP-OEC group (Table 2). Additionally, the total cell number counts of blastocysts exhibited non-significant differences across all groups, indicating that co-culture with OECs, regardless of hormone treatment, did not significantly affect blastocyst cell proliferation (Figures 2A, B). These results suggest that OEC co-culture significantly enhanced embryonic cleavage and promoted the formation of more blastocysts, with OECs treated with E2 and P4 demonstrating a more pronounced effect. However, it had a non-significant effect on the total cell number of blastocysts.

**TABLE 2:** Embryo development in the control group and co-culture of EP-OECs and OECs.

Group	No. of 4-cell	No. of 8-cell	No. of morula	No. of cleavages	No. of blastocysts	No. of embryos	No. of cleavage (%)	No. of blastocysts (%)
Control	16	18	62	131	28	151	86.8 (86.8 ± 0.01)^a^	18.6 (18.6 ± 0.01)^a^
EP-OEC	13	21	57	142	46	151	94.1 (94.1 ± 0.03)^b^	30.5 (30.5 ± 0.03)^b^
OEC	14	24	53	132	37	151	87.4 (87.4 ± 0.01)^a^	24.5 (24.5 ± 0.01)^c^

Cleavage and blastocyst formation rates (%) in control (no co-culture), EP-OEC, and OEC groups. Data: mean ± SEM. Cleavage rate: cleavage/total embryos. Blastocyst rate: blastocyst/total embryos. Values with different superscripts.

^a,b,c^ in the same column are significantly different (*p* < 0.05). Three replicates and the minimum number of oocytes in each replicate were 50. Control: SNCT embryos without co-culture treatment. EP-OECs: SCNT embryos co-cultured with oviduct epithelial cells treated with estradiol (E2) and progesterone (P4). OECs: SCNT embryos co-cultured with oviduct epithelial cells without hormone treatment.

### Effect of OEC co-culture on SCNT embryo quality

**FIGURE 3:**
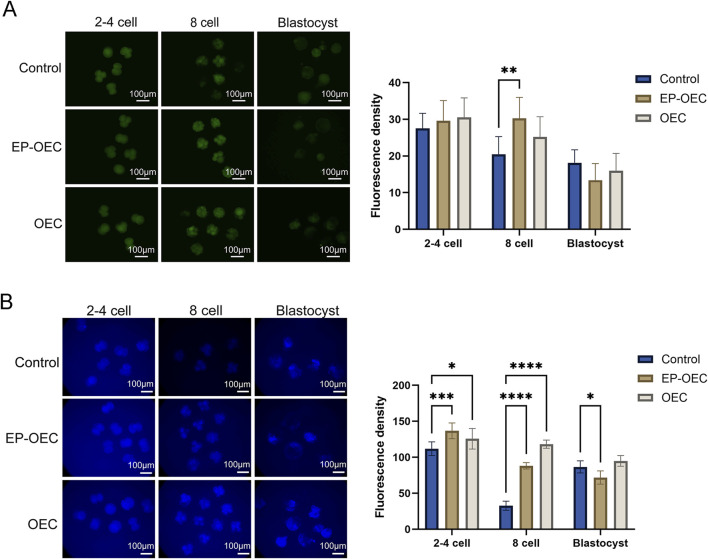
Intracellular ROS and GSH levels in SCNT embryos. **(A)** Intracellular ROS levels in SCNT embryos at the 2–4-cell, 8-cell, and blastocyst stages. **(B)** Intracellular GSH levels in SCNT embryos at the corresponding stages. Scale bar, 100 μm. The data are the mean ± SEM for the indicated gray value. *p < 0.05, **p < 0.01, and ***p < 0.001. Control, SCNT embryos cultured without co-culture. EP-OECs, SCNT embryos co-cultured with oviduct epithelial cells treated with estradiol (E2) and progesterone (P4). OECs, SCNT embryos co-cultured with oviduct epithelial cells without hormone treatment.

To explore the effects of co-culture with OECs or EP-OECs on SCNT embryo quality and antioxidant properties, the embryo intracellular ROS and GSH levels were measured at the 2–4 cell, 8-cell, and blastocyst stages (Figure 3). At the 2–4 cell stage, embryos co-cultured with OECs and EP-OECs exhibited slightly higher (non-significant) ROS levels than those in the control (Figure 3A). However, SCNT embryo co-culture with EP-OECs had a significantly higher ROS level at the 8-cell stage (Figure 3A); however, the OEC group did not exhibit this. At the blastocyst stage, there were no significant differences between the three groups.

Conversely, GSH levels in the EP-OEC and OEC groups were significantly higher than those in the control group at the 2–4 and 8-cell stages (Figure 3B). However, at the blastocyst stage, GSH levels in the EP-OEC group were significantly lower than those in the control group, whereas those in the OEC group were slightly higher but non-significantly different (Figure 3B). These results suggested that the increased metabolism of EP-OEC co-culture treatment further induced a higher ability to maintain redox balance. Such dynamic redox modulation may mimic the physiological oviductal conditions and contribute to improved embryonic developmental competence.

### Effects of the co-culture on gene expression patterns in the embryo

**FIGURE 4:**
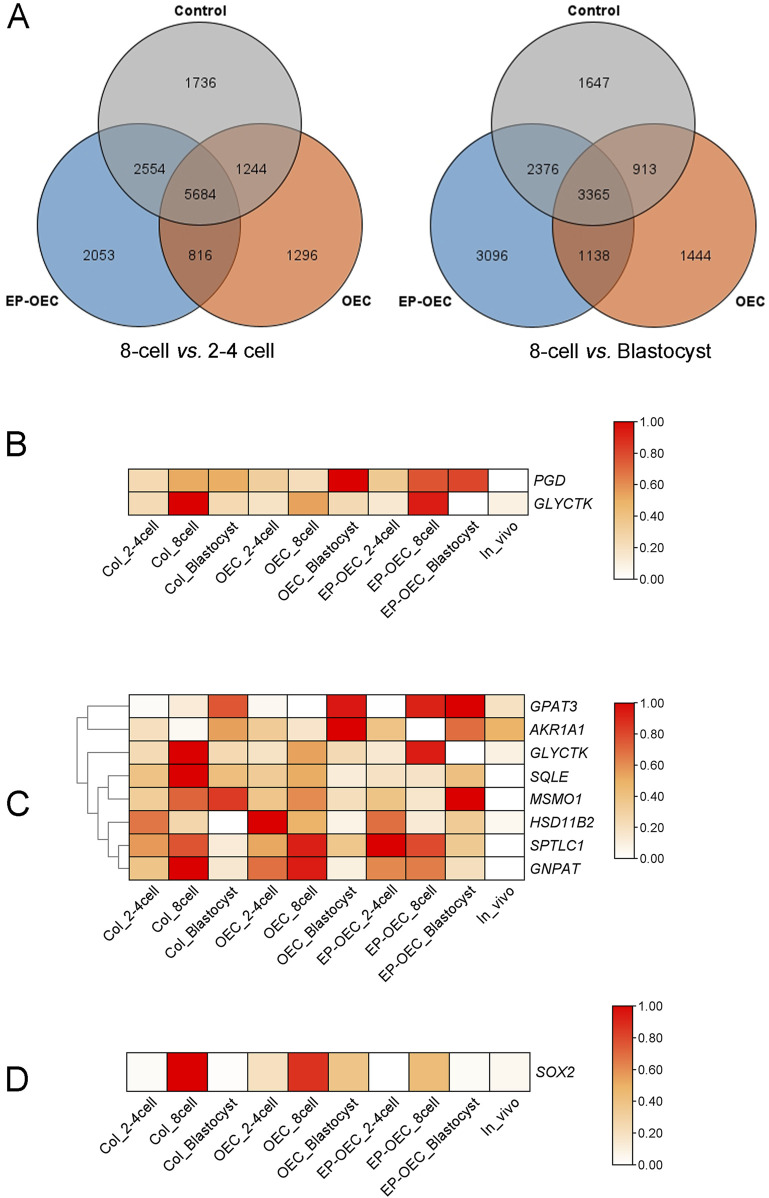
Transcriptomic profiling of SCNT embryos under different co-culture conditions. **(A)** Venn diagrams illustrating the overlap of differentially expressed genes (DEGs) at distinct developmental transitions: 8-cell vs. 2–4-cell (the left image) and 8-cell vs. blastocyst (the right image) stages in the control, OEC, and EP-OEC co-culture groups. **(B)** Heatmap showing the expression patterns of *PGD* and *GLYCTK*. **(C)** Heatmap of lipid metabolism-related DEGs. **(D)** Heatmap showing the relative expression of *SOX2*. The redder the color, the higher the level of gene expression; DEG, differentially expressed gene. Control, SCNT embryos cultured without co-culture. EP-OECs, SCNT embryos co-cultured with oviduct epithelial cells treated with estradiol (E2) and progesterone (P4). OECs, SCNT embryos co-cultured with oviduct epithelial cells without hormone treatment.

In this study, 2–4-cell stage embryos (shortened as 2–4 cells), 8-cell stage embryos (shortened as 8-cell), blastocysts, and *in vivo-*derived embryos were used for Smart-seq RNA-seq. A total of 267.88 Gb of high-quality sequence data were obtained from the NovaSeq platform. By analyzing DEGs from the comparisons of 8-cell versus 2–4-cell stages and blastocyst versus 8-cell stage under different co-culture conditions, we identified 15,383 and 13,979 DEGs, respectively (Figure 4A). Further analysis revealed that a subset of genes was differentially expressed in the 8-cell stage versus 2–4-cell stages and blastocyst stage versus 8-cell stage comparisons. Subsequent KEGG pathway enrichment analysis revealed that the “pentose phosphate pathway” was significantly enriched in the OEC and EP-OEC groups. Specifically, genes within the “pentose phosphate pathway” exhibited altered expression levels under co-culture conditions. The OEC co-culture treatment upregulated *PGD* (ENSSSCG00000003402) and *GLYCTK* (ENSSSCG00000011432) (Figure 4B). Furthermore, co-culture with hormone-treated OECs significantly disrupted the “lipid metabolism pathway” in embryos. Genes involved in lipid metabolism exhibited different expression patterns in the different groups (Figure 4C).

To compare the differences between EP-OECs and OECs, we analyzed the DEGs across experimental groups at identical developmental stages and observed 1,277, 1,240, and 1,229 DEGs exhibiting exclusive differential expression in the EP-OEC group at the 2–4-cell, 8-cell, and blastocyst stages, respectively. Among them, 37 and 50 genes had higher and lower expression levels, respectively, in EP-OECs. Among the 37 DEGs, the expression levels of *KAT8*, *PLIN2*, and *CYP2D6* were high, which were greatly affected by EP-OEC co-culture (Figure 4D). We also identified that among the 50 downregulated genes, *SOX2* was significantly affected by the EP-OEC co-culture treatment (Figure 4D).

**FIGURE 5:**
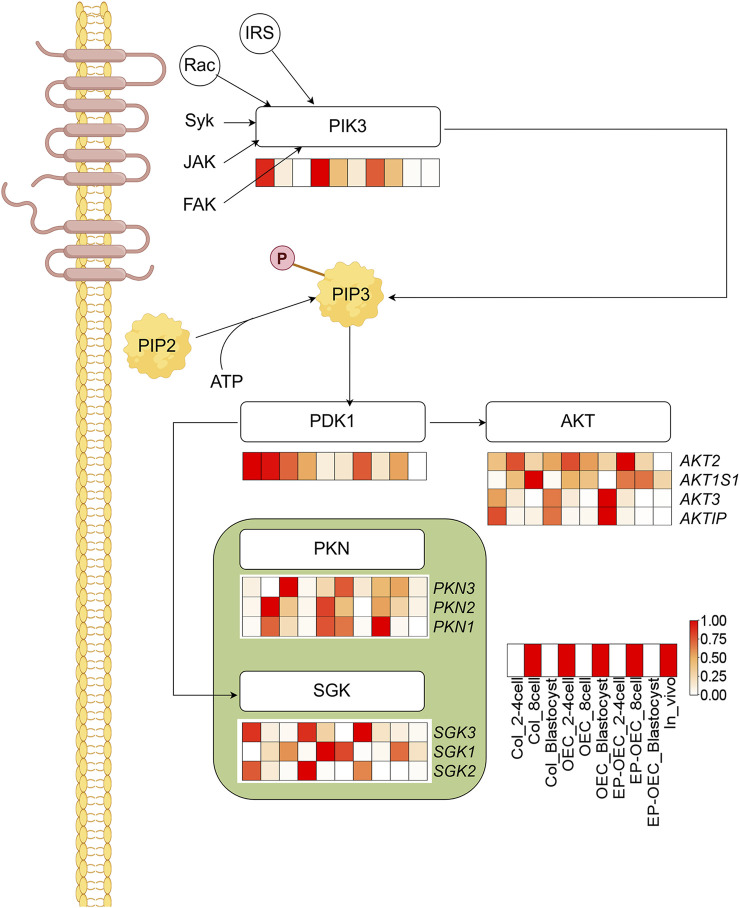
Enrichment of the PI3K-AKT signaling pathway in SCNT embryos. KEGG analysis identified significant enrichment of DEGs in the PI3K-AKT pathway. Heatmaps show expression patterns of key genes. The redder the color, the higher the level of gene expression; DEG, differentially expressed gene. Control, SCNT embryos cultured without co-culture. EP-OECs, SCNT embryos co-cultured with oviduct epithelial cells treated with estradiol (E2) and progesterone (P4). OECs, SCNT embryos co-cultured with oviduct epithelial cells without hormone treatment.

We further performed KEGG enrichment analysis on DEGs among the three groups and identified that multiple DEGs were significantly enriched in the “PI3K-AKT signaling pathway”, with particular attention warranted for *PIK3R3* attention. A schematic representation of the “AMPK signaling pathway” was generated, and the expression levels of key genes within the pathway were depicted using heatmaps (Figure 5). Comparative analysis revealed that *PIK3* and *PDK1* exhibited significantly elevated expression in the OEC co-culture groups compared to the control group.

## Discussion

SCNT in swine has become a valuable approach for producing gene-edited animals; however, its efficiency remains substantially lower than that in other species. This limited efficiency is primarily due to incomplete nuclear reprogramming and suboptimal *in vitro* culture conditions, which inadequately mimic the maternal reproductive tract environment. Consequently, swine SCNT embryos often display impaired developmental progression, reduced blastocyst formation rates, and poor survival of cloned offspring. Given the pivotal role of the oviduct in regulating early embryogenesis *in vivo*, recreating a more physiologically representative microenvironment *in vitro* has emerged as a crucial strategy for enhancing the developmental competence of swine SCNT embryos.

Under natural conditions, mammalian embryos interact with OECs and their secreted factors while passing through the isthmus, thereby enhancing their developmental potential (Fernandez-Fuertes et al., 2018). Previous studies have reported that co-culture with OECs can promote embryo development through a combination of nutritional support and regulation of molecular secretion (Xue et al., 2024; Choi et al., 2024). Based on this evidence, we selected OECs to mimic the *in vivo* microenvironment of early embryonic development and hypothesized that co-culturing swine SCNT embryos with OECs would significantly enhance their *in vitro* developmental competence.

The accurate identification and functional verification of OECs provided a cellular foundation for this study. Morphological evaluation confirmed that the primary OECs obtained were distinguishable from the PFFs. OVGP1 is known to be present during fertilization and early embryonic development in multiple mammalian species (Coy and Yanagimachi, 2015). In pigs, OVGP1 is expressed in the oviduct epithelium and is localized in the zona pellucida, perivitelline space, ovulated oocytes, and plasma membrane of oviductal embryos (Buhi et al., 1993). As the major mucin secreted by OECs, OVGP1 is vital for modulating the oviductal environment (González-Brusi et al., 2020). Immunofluorescence analysis confirmed strong OVGP1 expression in OECs, whereas no signal was detected in PFFs used as a negative control. Western blot showed that the expression of OVGP1 in the EP-OEC group was almost twice that in the control group. During the estrous cycle, the oviduct exhibits high sensitivity to fluctuations in E2 and P4 levels, with the expression of the hormone receptors PGR and ESR1 increasing during the simulated estrus phase (Maillo et al., 2016; Chen et al., 2018). The observed elevation in the expression of these genes in EP-OECs may reflect the activation of the secretory activity characteristic of the peri-ovulatory oviduct (Chen et al., 2013). Experimentally, EP-OECs displayed significantly enhanced *OVGP1* immunoreactivity and nearly twofold higher *OVGP1*, *ESR1*, *and PGR* mRNA levels than untreated OECs. These cyclic functional changes are likely to optimize reproductive processes within the oviduct. Given previous reports that OVGP1 can facilitate embryo development (Zhao et al., 2022), our findings suggest that hormone-pretreated OECs recapitulate the endocrine state of the peri-ovulatory oviduct more closely, thereby providing a microenvironment for embryo culture that is more physiologically relevant.

Co-culture with OECs significantly enhanced blastocyst formation efficiency (Lee et al., 2018), as demonstrated in this study. Specifically, OEC treated with E2 and P4 exhibited greater capacity to promote embryonic development (blastocyst formation rates: EP-OEC: 30.5%; OEC: 24.5%). The superior performance of EP-OECs may be attributed to estradiol and progesterone pretreatment, which likely stimulates the secretion of OVGP1 and other bioactive molecules and extracellular vesicles enriched in developmental regulatory factors, thereby increasing the availability of growth factors and supportive molecules that promote embryonic cell proliferation and differentiation (Harris et al., 2020; Bang et al., 2023), which is consistent with previous studies in cattle and sheep (Pranomphon et al., 2024; Davachi et al., 2017). Notably, blastocysts derived from the OEC and EP-OEC co-culture groups exhibited a total cell number comparable to that of the control, suggesting a limited impact on this metric of embryonic developmental quality. Furthermore, embryos in the co-culture system exhibited significantly elevated GSH levels compared with those in controls.

Previous evidence confirms that modulating intracellular GSH/redox status directly enhances preimplantation embryo quality (Abdul Rahman et al., 2022). Concurrently, embryos co-cultured with EP-OECs exhibited significantly elevated ROS levels at the 8-cell stage compared to controls, suggesting enhanced metabolic activity in this treatment group. Each critical step of early embryonic development requires finely tuned energy and metabolic adjustments that are largely dependent on the mitochondrial content and oocyte activity (May-Panloup et al., 2021). Mitochondria are the primary source of adenosine triphosphate and generate ROS, including hydrogen peroxide, superoxide, and hydroxyl radicals, during oxidative phosphorylation (Deluao et al., 2022). The elevated ROS levels observed in embryos from the 2-cell to the 8-cell stage may reflect the increased metabolic rate induced by the co-culture, including enhanced mitochondrial oxidative phosphorylation. High metabolic activity inevitably increases electron transport chain flux, thereby elevating ROS production. GSH is a major non-enzymatic intracellular antioxidant that directly scavenges ROS and maintains the cellular redox state (Lin and Wang, 2021). The co-culture environment may activate the endogenous antioxidant system of embryos, leading to the upregulation of genes involved in GSH synthesis. Although the ROS levels increased, the concomitant increase in GSH suggests that embryos maintained an effective redox balance, thus avoiding oxidative damage.

Comparative transcriptomic analysis revealed numerous DEGs across developmental stages and co-culture conditions, indicating extensive molecular remodeling during swine preimplantation development. The KEGG pathway enrichment analysis revealed significant enrichment of the pentose phosphate pathway (PPP) in the OEC and EP-OEC groups, with *PGD* and *GLYCTK* markedly upregulated in OEC co-cultures. PPP is vital for generating NADPH for reductive biosynthesis and maintaining redox homeostasis, processes that are particularly critical for SCNT embryos (Wen et al., 2020). Notably, EP-OECs induced broader transcriptomic alterations, particularly in lipid metabolism pathways. Changes in lipid metabolism are closely associated with membrane fluidity, energy storage, and production of signaling molecules during embryogenesis (Khan et al., 2021). The observed differential regulation suggests that EP-OECs may reprogram embryonic lipid utilization by secreting lipid-binding proteins or modulating lipid metabolism through hormonal signaling. The pronounced upregulation of *KAT8*, *PLIN2*, and *CYP2D6* in EP-OEC co-cultures is particularly noteworthy. *KAT8*, a histone acetyltransferase, mediates H4K16 acetylation and promotes chromatin relaxation and transcriptional activation (Cao et al., 2017). *PLIN2* encodes a lipid droplet-associated protein that is critical for lipid storage and mobilization, potentially enhancing the metabolic support for developing embryos (Zhu et al., 2023). *CYP2D6*, a cytochrome P450 enzyme, participates in the metabolism of endogenous compounds, including steroid hormones, which may fine-tune the local hormonal microenvironment (Chen et al., 2008). Collectively, these changes indicate that OECs provide a more complex metabolic and epigenetic remodeling capacity under hormonal stimulation.

*SOX2* is a member of the SRY-related HMG-box family of transcription factors and is a core pluripotency factor (Hagey et al., 2022). *SOX2* knockout in embryos results in peri-implantation lethality, and its deletion in ESCs results in the loss of self-renewal, with the cells becoming trophoblast-like stem cells (Tremble et al., 2021). In this study, *SOX2* expression was increased by OEC but decreased by EP-OEC at the 8-cell stage. Interestingly, *SOX2* downregulation did not reduce the efficiency of embryonic development but promoted it, suggesting that co-culture with hormone-primed OECs synergistically interacts with *SOX2* during embryonic development. The co-culture of EP-OECs compensates for the negative effect of *SOX2* downregulation.

Furthermore, *SOX2* expression was negligible at the blastocyst stage. The lower expression of *SOX2* levels observed during the 8-cell stage in EP-OEC co-cultured embryos may correlate with their expedited developmental progression. This mechanism is interesting and complex, and further work is needed to verify this. *SOX2* functions within complex regulatory networks involving other pluripotency factors, primarily *OCT4* and *NANOG*. In the absence of *SOX2*, these factors may partially compensate to sustain a certain level of pluripotency (du Puy et al., 2011).

After fertilization, the PI3K/AKT pathway is activated by autocrine trophic ligands, and this pathway is essential for early embryo development (Kalous et al., 2023). In early *Drosophila* embryos, *AKT* regulates centrosome migration and mitotic spindle orientation and promotes proper spindle morphology (Buttrick et al., 2008). *AKT* is vital for zygotic genome activation of the 2-cell embryo in mice (Chen et al., 2016). Moreover, *AKT* inhibition compromised the development of mouse embryos to the blastocyst stage, suggesting that *AKT* activity significantly affects normal blastocyst development (Li et al., 2007). It has been proposed that the regulation of blastomere proliferation in pre-implantation mouse embryos is based on *AKT* activity. Activated *AKT* is essential for mouse blastocyst formation and is indispensable for the first cell lineage differentiation in early mouse embryos (Kalous et al., 2023). Activation of the PI3K/AKT pathway promotes early embryonic development and facilitates the formation of the inner cell mass (ICM) in pigs (Oh et al., 2025; Jiao et al., 2020). Moreover, this signaling pathway plays an important role in trophoblast cell migration (Jeong et al., 2016). In this study, *PIK3* expression decreased with embryo development. At the 2–4-cell stages, the *PIK3* expression level in the EP-OEC group was the lowest; OEC and EP-OEC treatment promoted *PIK3* expression at the 8-cell stage.

Additionally, *PDK* was downregulated by the OEC and EP-OEC co-culture treatments. *AKT*, *PKN*, and *SGK* were also affected to varying degrees. The high expression of *PIK3* in OECs and EP-OECs suggested that the PI3K/AKT pathway is highly activated. Although the expression of *PIK3* in EP-OECs at the 2–4-cell stages was not high, this could not mask the positive effect of high expression at the 8-cell stage.

In summary, these transcriptomic findings provide a mechanistic framework for phenotypic improvements observed in EP-OEC co-cultured embryos. By influencing key metabolic pathways (PPP and lipid metabolism), modulating pluripotency-associated transcription factors (*SOX2*), and engaging central signaling cascades (PI3K–AKT and AMPK), hormonally regulated OECs appear to create a culture environment that closely approximates the *in vivo* oviduct. This environment supports oxidative and metabolic stability and fine-tunes the developmental timing, thereby increasing the likelihood of successful implantation and full-term development.

This study has made the above progress, but the current research lacks the data and mechanism analysis of post implantation development in pigs. It is essential to extend the research to the later stage with the support of *in vivo* validation and long-term analysis for a more comprehensive understanding of porcine embryogenesis.

## Conclusion

This study demonstrates that co-culture with OECs significantly enhances the *in vitro* developmental competence of swine SCNT embryos by closely mimicking the physiological oviduct microenvironment. E2- and P4-pretreatment potently activated OEC secretory functions, elevating key factors such as OVGP1 and optimizing the culture milieu. Mechanistically, co-culture with OECs/EP-OECs enhanced the metabolic activity while preserving the redox balance through increased GSH levels. Transcriptomic analysis revealed enrichment of critical pathways, such as the pentose phosphate pathway and PI3K–AKT signaling, and stage-specific modulation of pluripotency factors. OEC/EP-OEC co-culture treatment can significantly activate the PI3K–AKT signaling pathway activity in embryos, which may be a key mechanism that can promote embryonic development.

## Data Availability

The datasets presented in this study can be found in online repositories. The names of the repository/repositories and accession number(s) can be found in the article/supplementary material.
